# Pica in childhood: Prevalence and developmental comorbidity

**DOI:** 10.3389/frcha.2023.1099527

**Published:** 2023-02-08

**Authors:** Sigita Lesinskienė, Greta Stonkutė, Rokas Šambaras

**Affiliations:** ^1^ Faculty of Medicine, Institute of Clinical Medicine, Clinic of Psychiatry, Vilnius University, Vilnius, Lithuania; ^2^ Faculty of Medicine, Vilnius University, Vilnius, Lithuania

**Keywords:** children, internal and external difficulties, eating disorder, pica, preschool age, strengths and difficulties questionnaire, sensory sensitivity

## Abstract

**Background:**

Pica is an eating disorder in which a person feels the urge to eat non-nutritious, non-food substances. It can occur at any age; however, a higher prevalence is observed among children with mental health issues and pregnant women. Recently, additional attention has been given to the role of sensory sensitivity in eating disorders.

**Aim of the study:**

To examine the prevalence of pica in preschool children and explore the associations with increased sensory sensitivity traits and rates of internalizing and externalizing difficulties.

**Methodology:**

Parents/guardians of children aged 3–6 years were asked to complete an anonymous online questionnaire containing demographic data, questions regarding the peculiarities of children's eating, sensory sensitivity. Furthermore, a Strengths and Difficulties Questionnaire was also completed. This study included 655 participants. Of those 655, 41 study participants who did not complete the questionnaire were excluded. The final sample consisted of 614 participants who had completed the questionnaire.

**Results:**

Approximately, 3.7% of participants indicated that their child ate non-nutritious, non-food substances. Children with increased sensory sensitivity traits in response to sounds (*p* = 0.008), visual stimuli (*p* < 0.001), and skin contact (*p* = 0.006) ate significantly more non-nutritious non-food substances. Those who had higher scores on internalizing and externalizing difficulties had more difficulties associated with eating. Children who had increased sensory sensitivity to sounds, visual stimuli, or skin contact had significantly more internalizing difficulties (*p* < 0.05). There were no significant differences between higher externalizing difficulties and increased sensory sensitivity to sounds, visual stimuli, skin contact, or smells (*p* > 0.05).

**Conclusions:**

Children with increased sensory sensitivity traits were more likely to eat inedible substances than those without. Furthermore, children who had increased sensory sensitivity traits were significantly more picky about food and had more internalizing difficulties. It is important for specialists to combine clinical data on the characteristics of a child's development, including sensory, eating, and emotional health aspects.

## Introduction

One in eight young people have a likelihood of being diagnosed with at least one eating disorder by the age of 20, and although anyone regardless of age or gender can have these disorders, they are more commonly diagnosed in teenagers and young women (1). Pica is an eating disorder that is often associated with iron deficiency (2). Currently, the 10th version of the International Classification of Diseases (ICD-10-AM) distinguishes pica disorder as separate diagnoses with two codes: one of them belongs to other behavioral and emotional disorders that usually begin in childhood and adolescence (Code: F98.3 Pica in infancy and childhood), and the other is classified under other eating disorders and is for adults (Code: F50.8 Other eating disorders, pica in adults). In the 11th version of the International Classification of Diseases (ICD-11), which will be in use soon, pica is left with only one code for the disorder and no longer distinguishes between pica in adults and children, although the diagnostic criteria remain the same (3). Pica disorder is characterized by a persistent, compulsive urge to eat substances that are normally considered inedible, and importantly, that urge is not related to the individual's sociocultural customs and traditions (2). Pertinently, the urge to eat non-edible substances is not specific to a certain period of development, when it is common to do so until approximately 18–24 months and it is necessary that this behavior lasts for at least one month (2, 4). Pica is considered a health or life-threatening disorder, and various studies emphasize that if pica is suspected during a clinical examination of a patient, it is important for healthcare professionals to determine this early so that they are able to remove ingested substances quickly in order to avoid serious life-threatening complications (2, 5, 6). The prevalence of pica is currently unknown, and varies between studies due to different diagnostic criteria, study methodology, and the fact that it is often underdiagnosed (2). Pica is more common in individuals with autism spectrum disorder, attention deficit hyperactivity disorder, schizophrenia, obsessive-compulsive disorder, and depression (2, 5). It is also common in pregnant women; a meta-analysis of approximately 70 studies found that the overall prevalence of pica during pregnancy and postpartum is close to 30% (2). The etiology of pica itself is still not well understood. It is likely to be multifactorial, which is another reason why clinical assessment is difficult, contributing to the fact that pica is often underdiagnosed (2) Recently, scientific articles examining the etiology of eating disorders and aiming to improve their diagnosis have described the role of sensory sensitivity in eating disorders (7).

Recently, scientific articles can be found in the that mention the role of sensory sensitivity in eating disorders (7). That atypical sensory features are associated with the peculiarities of eating shows recently studied atypical sensory associations of sensory sensitivity characteristics with food selectivity in children with the autism spectrum disorder. Based on these studies, it is observed that children who are diagnosed with autism spectrum disorder, also more often had features of increased sensory sensitivity and therefore had difficulties with eating habits (8, 9). There are few studies on the link between pica disorder and people who have increased sensory sensitivity traits; however, recently, an increasing number of studies have examined these links (10). This is the first study of its kind in Lithuania on the topic of pica disorder. This study aimed to examine the prevalence of pica in preschool children and explore the associations with increased sensory sensitivity traits and rates of internalizing and externalizing difficulties of the Strengths and Difficulties Questionnaire (SDQ).

## Materials and methods

### Research participants

Research participants were recruited using non-probability convenience sampling, by inviting Lithuanian kindergarten parents/guardians of 3–6 year old children to fill out an online questionnaire. In the invitation to participate in the study, one of the parents/guardians was invited to answer a questionnaire about the child. The questionnaire was compiled by the author and their scientific supervisor. A total of 655 participants were included in this study. The study included data from people who completed the survey questionnaire and whose children were between three (36 months) and six (84 months) years of age. Of those 655, 41 study participants who did not complete the questionnaire were excluded. The final sample comprised 614 parents/guardians.

### Research instruments

The questionnaire contained questions regarding demographic data, children's eating habits, and increased sensory sensitivity. Furthermore, participants also completed the SDQ questionnaire.

The section on children's eating habits contained five closed-ended questions: (1) Does your child experience eating difficulties?; (2) Does your child eat independently?; (3) Does your child eat inedible things?; (4) Is your child very picky about food? Questions were answered by choosing one of two answer options: yes or no. Study participants who answered yes or do not know to the question about eating non-edible items were invited to record the non-edible items that the child eats.

The section on increased sensory sensitivity contained four questions: (1) Does your child have increased sensory sensitivity to sounds? (2) Does your child show signs of increased sensory sensitivity to visual stimuli?; (3) Does your child have increased sensory sensitivity to touch and skin sensitivity to clothing?; (4) Does your child have increased sensory sensitivity to odors? (5) Each question had two possible answers: yes or no.

The SDQ is a 25-item questionnaire assesses emotional problems, conduct problems, hyperactivity, peer problems, and prosocial behavior (11). Parents have rated the extent to which their child was characterized by SDQ items such as “Often has temper tantrums or hot tempers,” “Constantly fidgeting or squirming,” and “Often lies or cheats” on a 3-point scale: 0 (never), 1 (sometimes), or 2 (always). The SDQ-Total-Difficulties score has been calculated by summing the scores of all subscales, except for prosocial behavior. The SDQ-Internalizing Difficulties score has been calculated by summing the emotional and peer problems scores, and the SDQ-Externalizing Difficulties score has been calculated by summing the conduct problems and hyperactivity scores. The questionnaire has been previously validated in a Lithuanian-speaking population (12). The SDQ scores were analyzed as continuous variables, with higher scores indicating more difficulties. The Hebrew translation of the SDQ has been previously used in Lithuanian population (12). The internal consistency of the SDQ-Total Difficulties Score (Cronbach's alpha = 0.79) for the present study was sufficient.

### Procedure

The study comprised two phases. In the first phase, efforts were made to reach out to the study participants and recruit them using the snowball method. An invitation to contribute to the research was sent by e-mail to various preschool education institutions in Lithuania. When sending the questionnaire link, which was created using Google Forms, an invitation to participate in the study was attached, which also indicated how and who to contact with questions regarding the questionnaire or study. Those who agreed to participate in the study were invited to familiarize themselves with the description of the study, which indicated the purpose of the research and the people conducting it. After reading the descriptive text, the participants could move to the next part of the questionnaire, where they were invited to answer questions. In the second phase, those who agreed to participate were asked to fill out an online anonymous survey, which took approximately 20 min to complete. At any time, they could voluntarily withdraw from the study by discontinuing the questionnaire. In this case, the data was not used for the statistical analysis.

### Data analysis

The obtained data were coded manually using Microsoft Excel by assigning the corresponding numerical values to the corresponding answers. Microsoft Excel 2010 and IBM SPSS 26.0 programs were used for statistical data analysis. The results are presented as percentages, and continuous variables are expressed as means or medians with standard deviations. Pearson's *χ*^2^ and Fisher's criteria were used to assess the relationships between rank variables. The normal distribution of the data was assessed using Kolmogorov and Smirnov tests. Non-normally distributed groups of variables were evaluated for ranks using the Mann–Whitney *U* test for two samples.

## Results

### Participants

Of the participants, 594 (96.6%) were mothers/caregivers, and 21 (3.4%) were fathers/caregivers, with a mean age of 34.06 years (SD = 5.41; age range, 21–50 years). Further, 302 (49.2%) parents/guardians had higher university education, 126 (20.5%) had higher non-university education (graduated from college), 104 (16.9%) had received professional education, 71 (11.6%) completed high school, and 11 (1.8%) did not finish high school. Of the participants, 511 (83.2%) lived with a child and partner, 59 (9.6%) lived only with children, and 44 (7.2%) lived with children, partners, and other relatives. Finally, 301 (49.0%) indicated that they filled in the questionnaire for boys, and 313 (51.0%) filled it for girls. The average age of the children who the questionnaire was filled out for was 58.14 months (SD = 13.59; age range, 22–83 months). Most of the research participants filled out the questionnaire for children aged four (27.0%)- and five (26.4%)-year-old. Prevalence of eating difficulties in the study sample.

Of the 614 respondents, 134 (21.8%) indicated that their child had eating-related difficulties, and 480 (78.2%) said that their child did not have eating-related difficulties. There was no significant difference (*p* = 0.952) in the prevalence of eating difficulties between boys [*n* = 66 (21.9%)] and girls [*n* = 68 (21.7%)]. Most eating difficulties were observed among children aged 6 years (72–83 months) [*n* = 39 (28.1%)] (Table 1).

**Table 1:** Age distribution of children with eating difficulties, eating non-nutritious, non-food substances.

	Eating-related difficulties are present % (*n*)	Does not indicate eating-related difficulties % (*n*)
3 years (36–47 months)	25.8% (38)	74.2% (109)
4 years (48–59 months)	15.1% (25)	84.9% (141)
5 years (60–71 months)	19.8% (32)	80.2% (130)
6 years (72–83 months)	28.1% (39)	71.9% (100)
*p-value*	0.022	
	Eat non-nutritive, non-nutritive substances % (*n*)	Does not eat non-nutritive, non-nutritive substances % (*n*)
3 years (36–47 months)	5.4% (8)	94.6% (139)
4 years (48–59 months)	2.4% (4)	97.6% (162)
5 years (60–71 months)	6.2% (10)	93.8% (152)
6 years (72–83 months)	0.7% (1)	99.3% (138)
*p–value*	0.043	

Further, 589 (95.9%) said that their child could eat by themselves, and 25 (4.1%) that they did not eat on their own. A total of 23 (3.7%) participants noted that their children ate inedible substances. There was significant difference (*p = 0.008*) in the prevalence of eating inedible substances between boys [*n* = 5 (1.7%)] and girls [*n* = 18 (5.8%)]. After evaluating the eating of non-nutritious, non-food substances among age groups, it was found that the highest prevalence of this was among 5-year-old children (60–71 months) [*n* = 10 (6.2%)], (*p = 0.043*) (Table 1).

During the study, parents who indicated that their child was eating non-nutritious, non-food substances were asked to indicate the type of substance. Of all the options recorded by the participants, the most frequently recorded answer options were grouped, and the least frequent answer options that did not fall into the selected subgroups were highlighted in the “Other”. (1) Household items—toilet paper; (2) Stationery—pencils, plasticine, eraser, paper; (3) Other—toys, snow, underwear.

### Features of increased sensory sensitivity

The sensory sensitivities of the study sample were evaluated (Table 2).

**Table 2:** Descriptive statistics of increased sensory sensitivity traits in the total study sample.

	Have sensitivity % (*n*)	Does not have sensitivity % (*n*)
Sounds	13.8% (85)	86.2% (529)
Visual stimuli	4.1% (25)	95.9% (589)
Skin sensitivity	9.8% (60)	90.2% (554)
Smells	7.0% (43)	93.0% (571)

Regarding girls' sensory sensitivity, sensitivity to sounds [*n* = 44 (14.0%)] and skin sensitivity [*n* = 36 (11.5%)] were most often experienced. Among boys, the most sensitivity traits occurred in response to sounds [*n* = 41 (13.6%)]. No statistically significant differences were found between sex and the presence of increased sensory sensitivity traits. In comparison, a statistically significant relationship was found between eating non-nutritious, non-food substances and increased sensory sensitivity traits to visual stimuli and skin sensitivity (Table 3).

**Table 3:** Distribution of increased sensory (sensual) sensitivity traits based on sex and distribution of increased sensory sensitivities among those who eat non-nutritious, non-food substances and those who do not eat non-nutritious, non-food substances.

Increased sensory sensitivity traits	Boys *(n = 301) % (n)*	Girls *(n = 313) % (n)*	*p-*value	Eat non-nutritious, non-food substances *(n = 23) % (n)*	Does not eat non-nutritious, non-food substances (*n = 591*) *% (n)*	*p-*value
Sounds	13.6% (41)	14.0% (44)	*0.511*	17.4% (4)	13.7% (81)	*0.357*
Visual stimuli	3.3% (10)	4.8% (15)	*0.349*	13.0% (3)	3.7% (22)	*0.026*
Skin sensitivity	8.0% (24)	11.5% (36)	*0.144*	26.0% (6)	9.1% (54)	*0.018*
Smells	8.0% (24)	6.7% (19)	*0.259*	8.7% (2)	3.7% (22)	*0.225*

The prevalence of picky eating in the study sample was also determined. Of those who were picky eaters, 109 (47.8%) were boys, 119 (52.2%) were girls, (*p = 0.581*). Children who had increased sensory sensitivity in the skin [*n* = 32 (14.0%)] or smells [*n* = 21 (9.2%)] were significantly more picky (*p < 0.05*) about food than those who did not have increased sensory sensitivity (Table 4).

**Table 4:** Food pickiness and increased sensory sensitivity traits.

Increased sensory sensitivity traits	Picky eating *(n = 228) % (n)*	Non—picky eating *(n = 368) % (n)*	*p-value*
Sounds	17.5% (40)	12.3% (45)	*0.291*
Visual stimuli	3.5% (8)	4.6% (17)	*0.808*
Skin sensitivity	14.0% (32)	7.6% (28)	*<0.001*
Smells	9.2% (21)	5.9% (22)	*0.012*

### Results of strengths and difficulties questionnaire (SDQ)

Evaluating children according to the SDQ scores, the total average of the sample was 8.86 (SD = 4.75; median- 8.0, (minimum estimate - 0, maximum - 30). However, the data were not normally distributed (*p* < 0.001), so further results will be presented as the median and mean ranks.

The scores of the SDQ internalizing difficulties found that the average of the sample was 3.20 (SD = 2.64; median- 3.0 (minimum estimate - 0, maximum - 15). However, the data were not normally distributed (*p* < 0.001).

According to the scores of the SDQ externalizing difficulties, the average of the sample was 5.66 (SD = 3.04; median- 5.0, (minimum estimate - 0, maximum - 17). However, once again, the data were not normally distributed (*p* < 0.001). Children who had eating-related difficulties also had statistically significantly (*p < 0.05*) higher SDQ scores on both the total scale and the internalizing and externalizing difficulties scales. The distribution of SDQ results in children with and without eating-related difficulties is presented in Table 5.

**Table 5:** Distribution of SDQ results in children who have and who do not have eating-related difficulties.

	Eating-related difficulties reported as present *Mediana (Mean rank)*	Eating-related difficulties reported as absent *Mediana (Mean rank)*	*p-value*
Overall scale	10 (359.41)	8 (93.29)	*<0*.*001*
Internalizing difficulties	4 (350.06)	3 (295.85)	*0*.*002*
Externalizing difficulties	6 (353.17)	5 (294.99)	*0*.*001*

Children who had increased sensory sensitivity to sounds, visual stimuli, or skin contact had significantly higher scores for internalizing difficulties (*p* < 0.05). There were no statistically significant differences between higher externalizing difficulties and increased sensory sensitivity to sounds, visual stimuli, skin contact, or smells (*p* > 0.05) (Table 6).

**Table 6:** Sensory sensitivity traits and SDQ results.

	Sensitivity to sounds *Mediana (Mean rank)*		Sensitivity to visual stimuli *Mediana (Mean rank)*			Sensitivity to skin *Mediana (Mean rank)*		Sensitivity to smells *Mediana (Mean rank)*	
	*present*	*absent*	*present*	*absent*		*present*	*absent*	*present*	*absent*
Overall scale	10 (376.52)	8 (296.56)	11 (411.92)	8 (303.25)		11 (393.60)	8 (298.82)	9 (359.13)	8 (303.71)
*p*	*0.001*		*0.003*			*0.001*		*0.050*	
Internalizing difficulties	4 (400.64)	2 (292.74)	5.5 (430.23)		3 (302.51)	4.5 (422.66)	3 (295.49)	4 (366.21)	3 (303.19)
*p*	*0.001*		*0.001*			*0.001*		*0.025*	
Externalizig difficulties	5 (327.57)	5 (304.32)	5.5 (342.77)		5 (306.07)	6 (343.93)	5 (303.70)	5.5 (331.24)	5 (305.76)
*p*	*0.262*		*0.317*			*0.098*		*0.366*	

## Discussion

The results of the study showed that 3.7% of participants indicated that their child ate non-nutritious, non-food substances. Prospective population-based cohort study reported that preschooler's pica behavior at 38 and 54 months were 2.3% and 0.7% (13). Also, in a similar study, the prevalence of pica among preschoolers (30 to 68 months of age) was 3.5% (5). However, it is worth noting that in both studies, the prevalence of pica was observed in several groups of preschoolers: (1) Children who have an autism spectrum disorder or intellectual disability. (2) A general population-based control group. Both studies clarified that Pica is more common in young children with autism spectrum disorder or intellectual disability. In our study, the number of preschoolers who have an autism spectrum disorder or intellectual disability is not known. Therefore, a direct comparison of pica prevalence is not fully correct.

In our study a statistically significant relationship was found between eating non-nutritious, non-food substances and increased sensory sensitivity traits to visual stimuli and skin sensitivity. Based on the literature, sensory processing difficulties, and especially when it comes to tactile sensitivity, are commonly reported in children with Autism disorder and may result in atypical eating and pica behavior (14).

Also the results of the study showed that girls were significantly more likely to eat non-nutritious, non-food substances than boys. Unfortunately, there is relatively little scientific research on these topics, especially regarding preschool children. It can be hypothesized that if a larger sample was interviewed, the proportion of girls and boys would be more similar.

Children who had eating-related difficulties had statistically significantly higher SDQ scores on both the total scale and the internalizing and externalizing difficulties scales. There are few studies on the link between pica disorder and autism spectrum disorder, attention deficit hyperactivity disorder, obsessive-compulsive disorder, and depression (2, 5). These disorders generally reveal higher scores on the SDQ scale (15).

The questions presented in the questionnaire focused on the characteristics of common eating in the group of children aged 3–6 years, but these were focused specifically on sensory sensitivity and eating inedible substances. In future research, it would be important to include questions regarding the frequency, duration, and circumstances of eating non-nutritious, non-food substances. Research into the prevalence, diagnosis, and treatment of pica disorder is important because, in addition to being potentially life-threatening, it is also a disorder that can potentially lead to social stigma owing to its relatively distinct symptoms, and thus lead to additional emotional stress for a person (6). There is relatively little scientific research on these topics, therefore these themes need to be explored further. The novelty and strength of this study is that it examines preschool populations at kindergarten level and not developmentally disordered children.

Currently, there are no scientifically proven causes for the appearance of pica, and it is believed that this disorder may be determined by several factors (2). As the complications of this disorder can be life threatening, it is important that pica disorder be recognized and diagnosed as early as possible, so that healthcare professionals who may encounter pica are able to assess and recognize the risk factors (16). In a patient's account of their medical history it is important to pay attention to whether the patient has any other disorder, and when collecting social information during the anamnesis, one should determine the economic situation of the family, and assess whether the patient experiences neglect or abuse (2, 5, 6, 10). There is a possibility that when taking an anamnesis, there will remain unexplained symptoms or signs that may signal a pica disorder, as it largely depends on the patient themselves and/or parents/guardians provision of information who may deliberately withhold information. Therefore, it is important for an interdisciplinary team to work with the patient, as it increases the likelihood that specialists will notice or pay attention to the signs or symptoms, and perform a more thorough examination and evaluation (6). As the etiology and pathogenesis of pica have not yet been elucidated, there is no current pharmacological treatment that can be applied; however, research shows that behavioral therapy is an effective way to treat pica (17, 18).

### Limitations

The study participants were not clinically tested; thus, the obtained research results may not be an accurate diagnosis of pica. Furthermore, DSM 5 definition of pica includes a frequency criteria (A one), which is “Persistent eating of non-nutritious, non-food substances over a period of at least 1 month”. In our questionnaire the question is “does your child eat inedible things” without any reference to the persistence of the behaviour or follow up questions. The risk is to confuse a behavior with a disorder. It is acknowledged that the use of an opt-in volunteer sample may have biased study participants' engagement in the study. It may be that parents who are more concerned about their children's eating difficulties and pica are more likely to respond, or conversely less likely due to perceived stigma.

Future studies could continue the research on pica disorder in clinical samples. It is important to conduct longitudinal studies and observe how the consumption of non-nutritious, non-food substances changes throughout childhood development, which this study did not look at. Future research may benefit from tracking and assessing changes to these behaviors over time.

## Conclusions

Children with increased sensory sensitivity traits were more likely to eat inedible substances than those without. Furthermore, children who had increased sensory sensitivity traits were significantly more picky about food and had more internalizing difficulties. It is of importance for specialists to combine clinical data on the characteristics of a child's development, including sensory, eating, and emotional health aspects.

## Data Availability

The raw data supporting the conclusions of this article will be made available by the authors, without undue reservation.

## References

[1] Balasundaram P Santhanam P. Eating disorders. In: P Balasundaram, editor. Statpearls. Treasure Island, FL: StatPearls Publishing (2022). p. 6–14. Available from: https://www.ncbi.nlm.nih.gov/books/NBK567717/ (Accessed December 15, 2022). 33620794

[2] Leung AKC Hon KL. Pica: a common condition that is commonly missed—an update review. Curr Pediatr Rev. (2019) 15:164–9. 10.2174/1573396315666190313163530 30868957

[3] World Health Organization. ICD-11 for Mortality and Morbidity Statistics (2018). Available from: https://icd.who.int/browse11/l-m/en (Accessed December 18, 2022).

[4] American Psychiatric Association. Diagnostic and statistical manual of mental disorders fifth edition (DSM-5). Washington, DC; London, England: American Psychiatric Publishing (2013). 675–8 p.

[5] Fields VL Soke GN Reynolds A Tian LH Wiggins L Maenner M Pica, autism, and other disabilities. Pediatrics. (2021) 147(2):e20200462. 10.1542/peds.2020-0462 PMC9188765 33408069

[6] McNaughten B Bourke T Thompson A. Fifteen-minute consultation: the child with pica. Arch Dis Child Educ Pract Ed. (2017) 102(5):226–9. 10.1136/archdischild-2016-312121 28487433

[7] Sim L Peterson CB. The peril and promise of sensitivity in eating disorders. Int J Eat Disord. (2021) 54:2046–56. 10.1002/eat.23606 34536033

[8] Chistol LT Bandini LG Must A Phillips S Cermak SA Curtin C. Sensory sensitivity and food selectivity in children with autism spectrum disorder. J Autism Dev Disord. (2018) 48:583–91. 10.1007/s10803-017-3340-9 29116421 PMC6215327

[9] Thye MD Bednarz HM Herringshaw AJ Sartin EB Kana RK. The impact of atypical sensory processing on social impairments in autism spectrum disorder. Dev Cogn Neurosci. (2018) 29:151–67. 10.1016/j.dcn.2017.04.010 28545994 PMC6987885

[10] Al Nasser Y Muco E Alsaad AJ. Pica. In: Y Al Nasser, editor. Statpearls. Treasure Island, FL: StatPearls Publishing (2022). p. 1–7. Available from: https://www.ncbi.nlm.nih.gov/books/NBK532242/ (Accessed November 17, 2022). 30335275

[11] Goodman R Ford T Simmons H Gatward R Meltzer H. Using the Strengths and Difficulties Questionnaire (SDQ) to screen for child psychiatric disorders in a community sample. Br J Psychiatry. (2000) 177:534–9. 10.1192/bjp.177.6.534 11102329

[12] Gintilienė G Girdzijauskienė S Černiauskaitė D Lesinskienė S Povilaitis R Pūras D. A standardised Lithuanian version of Strenghts and Difficulties Questionnaire (SDQ) for school-aged children. Psichologija (Vilniaus Univ). (2004) 29:88–105. 10.15388/Psichol.2004.4355

[13] Emond A Emmett P Steer C Golding J. Feeding symptoms, dietary patterns, and growth in young children with autism spectrum disorders. Pediatrics. (2010) 126:337–42. 10.1542/peds.2009-2391 20643716

[14] Provost B Crowe TK Osbourn PL McClain C Skipper BJ. Mealtime behaviors of preschool children: comparison of children with autism spectrum disorder and children with typical development. Phys Occup Ther Pediatr. (2010) 30:220–33. 10.3109/01942631003757669 20608859

[15] Muris P Meesters C van den Berg F. The Strengths and Difficulties Questionnaire (SDQ)–further evidence for its reliability and validity in a community sample of Dutch children and adolescents. Eur Child Adolesc Psychiatry. (2003) 12:1–8. 10.1007/s00787-003-0298-2 12601558

[16] Rajput N Kumar K Moudgil K. Pica an eating disorder: an overview. Pharmacophore. (2020) 11:11–4.

[17] Moline R Hou S Chevrier J Thomassin KA. Systematic review of the effectiveness of behavioral treatments for pica in youths. Clin Psychol Psychother. (2020) 28:39–55. 10.1002/cpp.2491 32628326

[18] Ledford JR Barton EE Rigor MN Stankiewicz KC Chazin KT Harbin ER Functional analysis and treatment of pica on a preschool playground. Behav Anal Pract. (2018) 12:176–81. 10.1007/s40617-018-00283-9 30918781 PMC6411548

